# A Bayesian Sample Size Estimation Procedure Based on a B-Splines Semiparametric Elicitation Method

**DOI:** 10.3390/ijerph192114245

**Published:** 2022-10-31

**Authors:** Danila Azzolina, Paola Berchialla, Silvia Bressan, Liviana Da Dalt, Dario Gregori, Ileana Baldi

**Affiliations:** 1 Unit of Biostatistics, Epidemiology and Public Health, Department of Cardiac Thoracic Vascular Sciences and Public Health, University of Padova, 35122 Padua, Italy; 2 Department of Environmental and Preventive Science, University of Ferrara, 44121 Ferrara, Italy; 3 Department of Clinical and Biological Sciences, University of Torino, 10124 Turin, Italy; 4 Department of Pediatrics, University of Padova, 35122 Padua, Italy

**Keywords:** Bayesian trial, semiparametric, elicitation, sample size, phase II

## Abstract

Sample size estimation is a fundamental element of a clinical trial, and a binomial experiment is the most common situation faced in clinical trial design. A Bayesian method to determine sample size is an alternative solution to a frequentist design, especially for studies conducted on small sample sizes. The Bayesian approach uses the available knowledge, which is translated into a prior distribution, instead of a point estimate, to perform the final inference. This procedure takes the uncertainty in data prediction entirely into account. When objective data, historical information, and literature data are not available, it may be indispensable to use expert opinion to derive the prior distribution by performing an elicitation process. Expert elicitation is the process of translating expert opinion into a prior probability distribution. We investigated the estimation of a binomial sample size providing a generalized version of the average length, coverage criteria, and worst outcome criterion. The original method was proposed by Joseph and is defined in a parametric framework based on a Beta-Binomial model. We propose a more flexible approach for binary data sample size estimation in this theoretical setting by considering parametric approaches (Beta priors) and semiparametric priors based on B-splines.

## 1. Introduction

Bayesian trials are increasingly popular in clinical research especially when studies are conducted in poor accrual settings or on rare diseases [1,2].

International guidelines suggest planning a trial at the preliminary stage when the study design is defined before analyzing the data [3]. It follows that the study design definition and the sample size computation play a fundamental role in a frequentist, but also in a Bayesian, context [4].

A frequentist clinical trial is generally designed to optimize the study power, defined as the probability of truly detecting a treatment effect. Therefore, the power function is strongly related to the number of patients involved in the study [5]. 

In several research settings, early phase, pediatric or rare disease trials, patient enrollment, and accrual can be a challenging issue; as a consequence, the smaller study size, as compared to what was proposed in the prespecified study protocol, leads to a loss in the study power [2]. 

A Bayesian design, instead, may be helpful for small sample size trials because the method uses the available information about treatment effects, translated into informative prior distributions, to reduce the uncertainty in the treatment effect size estimation instead of providing a definitive answer to a statistical hypothesis, as defined in a frequentist study design [2,6].

Different Bayesian methods are available in the literature to obtain a sample size estimation procedure for binary data based on optimization of the precision, defined on a generic posterior interval or the posterior variance. Some authors identify the sample size by defining a tolerance area, *R*, in which the parameters of a binomial or multinomial distribution will be contained with a specified probability [7]. For example, Pham-Gia and Turkkan obtained sample sizes for a binomial distribution, in closed form considering a Beta-Binomial model, by imposing precision conditions on the posterior variance and the Bayes risk factor [8]. 

Other precision approaches to sample size estimation are based on the optimization of the length and coverage of the HPD (highest posterior density) interval. This kind of posterior interval is based on the assumption that any point within the interval has a higher density than any other point outside the interval, including the most likely values of the parameters. However, HPD does not always result in an interval estimate when the posterior density is multimodal; then, HPD can yield non-interval set estimators, in contrast to a quantile credibility interval [9]. Widely adopted procedures among HPD sample size estimation methods are the average coverage criterion (ACC) [10], the average length criterion (ALC) [10], and the worst outcome criterion (WOC) [10]. 

The ACC fixes the length of the posterior intervals and controls the coverage probability level over the data. The ALC method instead fixes the coverage rate and optimizes the length of intervals among the data space. The WOC, a more conservative criterion, controls both the length and coverage of the intervals over all possible data [10]. The sample size is conservative if it is larger, keeping fixed other factors involved in the design, indicating a greater degree of confidence in identifying the treatment effect of interest [11]. However, in some cases, excessively large sample sizes are not necessary because a smaller number of patients may be useful in estimating the expected effect with the same precision. The tradeoff between a more and less conservative design depends on available prior information, agreement among experts, safety issues, etc [12].

Other generalizations of the HPD interval sample size approaches provided in the literature consider the median coverage and length of the intervals among the data space or perform WOC computations on a specific subset of data [13]. 

All the proposed sample size solutions are developed considering a parametric definition of the prior distribution, specifically in a Beta-Binomial framework for binary data [14]. 

The definition of an appropriate prior distribution plays a central role in Bayesian trial design and analysis [15]. The data retrieved by other studies may be considered to derive informative distributions (objective prior). However, in some cases, empirical evidence on treatment effect is unavailable; in this research setting, expert opinions may be translated into an informative prior (elicitation process) [16].

Different methods may be considered to conduct an elicitation procedure in a parametric, semiparametric, and nonparametric setting. The parametric methods force the expert’s opinion into a prespecified density function characterized by hyper-parameters [17].

Nonparametric elicitation does not make any assumption about the distribution form of the expert opinion, and semiparametric approaches are hybrid solutions [18]. The literature indicates that, in several cases, nonparametric or semiparametric approaches are more pliable for eliciting expert beliefs [17,19].

The B-splines semiparametric method, for example, is a very flexible procedure that leads to obtaining a prior distribution by performing a balanced optimization of a weighted sum of two components; one is a linear combination of B-splines adapted among experts’ quantiles, and another is an uninformative uniform prior distribution [18]. 

Recently, the literature has evidenced some efforts to incorporate alternative procedures to the prior definition during the study design phase. The method is tailored to a phase IIA trial and represents a Bayesian counterpart of a Simon two-stage design, using historical data and semi-parametric prior elicitation methods [20].

Instead, this work proposes a generalized version of the ALC, ACC, and WOC, including parametric approaches (Beta priors) and semiparametric priors based on B-splines in the sample size estimation method. 

The proposed sample size estimation method is also applied to a motivating example, a phase II clinical trial that assesses the effects of pharmacological treatment on a binary safety endpoint in a pediatric population. This kind of study design generally has one sample, is single-stage, and is conducted on small sample sizes, in which enrolled patients are treated and then observed for a possible response, generally binary [21]. The opinions provided by eight experts are considered to elicit informative priors used to design the trial in both a parametric Beta-Binomial and semiparametric B-splines setting.

## 2. Materials and Methods

### 2.1. Theoretical Setting

#### 2.1.1. Bayesian Methods and Criteria for Sample Size Estimation

Considering an unknown parameter θ, and a parametric space Θ for unknown  θ, the prior distribution has a density function  f(θ). The data, considering a sample size equal to *n*, are $x=\left(x_{1},x_{2},\ldots ,x_{n}\right)$ and are assumed interchangeable among data space *χ*.

Different Bayesian sample size criteria are proposed in the literature for binary data, as follows:

1. Average coverage criterion [7];
2. Average length criterion [10];
3. Worst outcome criterion [10].

Average Coverage Criterion. Considering an HPD credible interval, it is possible to assume a fixed length *l*. The coverage (1−α) instead varies with the data among the overall data space *χ*. An ACC sample size is the smallest integer such that, for a length *l*, the expected coverage level is at least  1−α.

$$\int_{\chi }\left\{\int_{a\left(x,n,l\right)}^{a\left(x,n,\;l\right)+l}f(\theta |x,n)d\theta \right\}g\left(x\right)dx\geq 1-\alpha$$

The predictive distribution of the data, commonly referred to as the preposterior marginal distribution, is  g(x); the posterior distribution is  f(θ|x), defined as combining likelihood information g(x|θ)  and prior distribution f(θ). In the equation, *a*(*x*, *n*, *l*) is the lower bound of the HPD interval of prespecified length *l*, considering a posterior density function f(θ|x,n) related to data *x* and sample size *n*. The relation reported on the left side of the equation may be interpreted as an average of the posterior coverage, weighted by the data’s predictive distribution *g*(*x*).

Average Length Criterion. The ACC defines the sample size, fixing the coverage probability (1−α) of the HPD interval. The first step, in this case, is to find in the data space the interval lengths $l^{\prime }\left(x,n\right)$ satisfying this condition

$$\int_{a\left(x,n,\;\alpha \right)}^{a\left(x,n,\alpha \right)+l^{\prime }\left(x,n\right)}f(\theta |x)d\theta =1-\alpha$$

The optimal sample size is the minimum integer that satisfies the condition:

$$\int_{x}l^{\prime }\left(x,n\right)g\left(x\right)dx⩽l$$

In this relation, *l* is the prespecified length. In this case, the left side of the equation is a mean of the lengths of the HPD intervals among the data space, weighted by the predictive distribution  g(x).

The solution to this equation is not necessarily an HPD interval; for this reason, it is essential to verify the conditions proposed in the literature to guarantee that a generic credibility interval is an HPD interval [22].

Worst Outcome Criterion. The WOC criterion fixes both coverage probability (1−α) and length *l* in advance, ensuring a specified length and a minimum coverage among data *x* in the data space.

The sample size may be found by choosing the minimum *n*, ensuring that:

$$\underset{x\in X}{\inf}\left\{\int_{a\left(x,n,\alpha ,l\right)}^{a\left(x,n,\alpha ,l\right)+l}f(\theta |x)d\theta \right\}⩾1-\alpha$$

Considering the case of the estimation for a single proportion, the relation on the left side of the equation becomes:

$$\int_{a\left(x,n,\alpha ,l\right)}^{a\left(x,n,\alpha ,\;l\right)+l}f(\theta |x,n,c)d\theta$$

This integral cannot be minimized for all the values of *n*, *c*, *d*, and *l*, where *c* is the expected number of successes. Therefore, some conditions have to be identified to find a subset of data x* as indicated in the literature [10].

$$\int_{a\left(x^{*},n,\alpha ,l\right)}^{a\left(x^{*},n,\alpha ,l\right)+l}f(\theta |x^{*},n,c)d\theta ⩾1-\alpha$$

#### 2.1.2. Semiparametric Method for Prior Elicitation

The prior distribution for proportions may be derived in a semiparametric approach, eliciting an expert opinion [18].

In this framework, the prior distribution is obtained by performing optimization of a weighted sum of the following two components:

1. A term assessing the goodness of fit of a prior distribution among expert quantiles
2. The distance of the prior respect to a uniform uninformative distribution.

A uniform distribution has been considered as an uninformative prior, as suggested in the literature [18], to obtain a prior distribution that is a flexible compromise between a full uninformative prior and an informative function adapted among expert quantiles.

The functional form of the probability density function among expert quantiles is approximated by a linear combination of B-splines with inner knots corresponding to specific boundaries. In this theoretical framework, *F* is a spline having *m* degree with a sequence of *S* inner knots $\lambda ={\left(\lambda_{-m},\ldots ,\lambda_{S+m+1}\right)}^{T}$. As indicated in the literature, some constraints have been imposed to guarantee that the linear combination is a density function [18].

Assuming it has *p*-elicited quantiles $y_{\alpha_{1}},\ldots ,y_{\alpha_{p}}$ modeled by a linear combination of B-splines, the expert density function may be determined by optimizing this objective function:

$$\underset{F_{-m},\ldots ,F_{S}}{\min}\left\{\overset{p}{\underset{i=1}{\sum }}{\left(\alpha_{i}-F\left(y_{\alpha_{i}}\right)\right)}^{2}+\phi \int_{y_{0}}^{y_{1}}f{\left(y\right)}^{2}dy\right\}\;F_{i}\leq F_{i+1}\;\mathrm{for}\;i=-m,\ldots ,S-1\;\mathrm{and}\;F_{-m}=0,F_{S}=1$$

where $F\left(y_{\alpha_{i}}\right)$ is the linear combination of B-splines (nonparametric component) stated among expert quantiles $\alpha_{i}$, the generic spline function defined by the *m* degree and the sequence of *S* inner knots is $F_{i}=F\left(\lambda_{i}\right)$, and $\int_{y_{0}}^{y_{1}}f{\left(y\right)}^{2}dy\;$ is a penalty term that measures the distance to the uniform distribution. Here, ϕ>0 is a balancing factor penalizing the distance between the function $F\left(y_{\alpha_{i}}\right)$, adapted to the expert quantiles $\alpha_{i}$ and the uniform distribution in the domain $\left[y_{0},y_{1}\right]$. Greater values of ϕ determine a prior distribution more similar to uniform, but instead, smaller values guarantee a posterior density that is better adapted among the expert quantile distribution.

This optimization problem may be easily solved using a quadratic programming method [18].

The balancing factor ϕ may be defined by fixing an expected Δ error reflecting the distance between an expert distribution and the stated *p* quantiles.

$$\Delta =\sqrt{\frac{1}{p}\overset{p}{\underset{i=1}{\sum }}{\left(\alpha_{i}-F\left(y_{\alpha_{i}}\right)\right)}^{2}}$$

This approach should define a realistic representation of the expert’s uncertainty. When such an uncertainty statement is not available, one might also use default values for Δ [18].

The authors suggest identifying the default Δ by considering a data-driven approach; the stated quantiles $y_{\alpha_{i}}$ have been normalized ($y_{\alpha_{i}{}^{*}}$) with respect to the domain bounds [j,k] of the parametric space under investigation.

$$y_{\alpha_{i}{}^{*}}=\left(y_{\alpha_{i}}-j\right)/\left(k-j\right)$$

The data-driven delta $\Delta^{*}$ is then calculated as the quadratic loss function of the stated standardized quantiles $y_{\alpha_{i}{}^{*}}$ and the numeric vector determining the levels of the quantiles $y_{i}$:

$$\Delta^{*}=\left(\sqrt{\frac{1}{p}\overset{p}{\underset{i=1}{\sum }}{\left(y_{\alpha_{i}{}^{*}}-y_{i}\right)}^{2}}\right)/2$$

#### 2.1.3. Semiparametric B-Splines Approach for Sample Size Estimation

The methods developed in a parametric setting for binary data (ACC, ALC, and WOC) sample size estimation are extended to incorporate the semiparametric B-splines priors obtained by eliciting experts’ opinions.

B-splines Average Coverage Criterion. The B-splines average coverage criterion (BSACC) sample size involves the same optimization problem as the ACC, incorporating a different prior distribution.

$$\overset{n}{\underset{x=0}{\sum }}Pr\left\{\theta \in \left(a\left(x,n\right),a\left(x,n\right)+l\right)\right\}p\left(x,n\right)⩾1-\alpha$$

In this framework, the coverage level may be reported as:

$$Pr\left\{\theta \in \left(a\left(x,n\right),a\left(x,n\right)+l\right)\right\}\propto \int_{a\left(x,n,l\right)}^{a\left(x,n,l\right)+l}\theta^{x}{\left(1-\theta \right)}^{\left(n-x\right)}f\left(\theta ,m,S,\phi ,y\right)d\theta$$

In this relation, f(θ,m,S,ϕ) is the prior distribution obtained with the B-splines method, depending on *m* degrees of approximation, *S* inner knots and ϕ  balancing factor and $y_{\alpha_{i}}$ expert quantiles

$$f\left(\theta ,m,S,\phi ,y\right)=\underset{F_{-m},\ldots ,F_{S}}{\min}\left\{\overset{p}{\underset{i=1}{\sum }}{\left(\alpha_{i}-F\left(y_{\alpha_{i}}\right)\right)}^{2}+\phi \int_{y_{0}}^{y_{1}}f{\left(y\right)}^{2}dy\right\}$$

In this context, the predictive preposterior distribution depending on data *x* is:

$$p\left(x,n\right)=\int_{\Theta }\theta^{x}{\left(1-\theta \right)}^{\left(n-x\right)}f\left(\theta ,m,S,\phi ,y\right)d\theta$$

The BSACC criterion becomes

$$\overset{n}{\underset{x=0}{\sum }}\int_{a\left(x,n,l\right)}^{a\left(x,n,l\right)+l}\theta^{x}{\left(1-\theta \right)}^{\left(n-x\right)}f\left(\theta ,m,S,\phi ,y\right)d\theta \int_{\Theta }\theta^{x}{\left(1-\theta \right)}^{\left(n-x\right)}f\left(\theta ,m,S,\phi ,y\right)d\theta ⩾1-\alpha$$

B-splines Average Length Criterion. The same minimization criterion has been provided for BSALC for a corresponding ALC. Considering the B-splines prior distribution setting, the length *l’(x*, *n)* may be found by solving:

$$\int_{a\left(x,n,\alpha \right)}^{a\left(x,n,\alpha \right)+l^{\prime }\left(x,n\right)}\theta^{x}{\left(1-\theta \right)}^{\left(n-x\right)}f\left(\theta ,m,S,\phi ,y\right)d\theta =1-\alpha$$

Once the candidate lengths in the data space have been found, the optimal sample size may be obtained by finding the minimum sample size such that:

$$\overset{n}{\underset{x=0}{\sum }}l^{\prime }\left(x,n\right)\int_{\Theta }\theta^{x}{\left(1-\theta \right)}^{\left(n-x\right)}f\left(\theta ,m,S,\phi ,y\right)d\theta ⩽l$$

B-splines Worst Outcome Criterion. The B-splines worst outcome criterion (BSWOC) may be found by imposing the same constraints as indicated in the WOC method for parametric solutions, imposing that the prior be adapted to an expert opinion in a unimodal function [10].

$$\int_{a\left(x^{*},n,\alpha \right)}^{a\left(x^{*},n,\alpha \right)+l}f\left(\theta |x^{*},m,S,\phi ,y\right)d\theta ⩾1-\alpha$$

### 2.2. Implementation

#### 2.2.1. Sample Size Estimation Procedure

A sample size estimation plan has been defined using a generalized ACC, ALC, and WOC estimation procedure (GACC, GALC, GWOC), which accounts for parametric prior distributions or semiparametric approaches based on B-splines defined on expert opinions. 

The method is generalized because it is based on HPD interval estimation among all data in the sample space, considering a wide range of prior distributions, not only a conventional Beta-Binomial parametric solution. 

The only constraint is that the prior distribution considered in the computation has to be unimodal. The HDI can also be computed for a distribution that is not severely multimodal; in this specific case, the HDI is the narrowest interval containing the specified mass. However, the computation does not always work properly for severely multimodal densities, where the HDI may be discontinuous. The single interval returned in this case may incorrectly include values between the modes with low probability density [23].

#### 2.2.2. Optimization Criteria

The optimal sample size has been identified by searching for the minimum integer optimizing the HPD interval length, coverage, or both criteria, by considering both parametric and semiparametric prior solutions.

#### 2.2.3. Interval Coverage and Length Optimization

For each sample size among all likelihoods in the overall data space, a Bayesian HPD (highest posterior density intervals) interval has been estimated; all the parameter values within HPD have a higher probability density than points outside the interval.

All intervals with a length equal to l (or with coverage equal to 1−α  for ACC) have been selected among the calculated HPD intervals.

Among the intervals, a weighted average of the coverages (or lengths for ALC) has been computed by weighting the posterior predictive distribution, which is the distribution for future predicted data based on the data already observed.

1. For the Beta prior case, the posterior predictive Is obtained in closed form as:

$$p\left(\tilde{x},n\right)=\int_{\Theta }\theta^{\tilde{x}}{\left(1-\theta \right)}^{\left(n-\tilde{x}\right)}Beta\left(a,b\right)d\theta \;=\left(\begin{array}{c} n\\ \tilde{x} \end{array}\right)\frac{B\left(a+\tilde{x},b+n-\tilde{x}\right)}{B\left(a,b\right)},\tilde{x}=0,\ldots ,n$$

B(a,b) is the Beta function for the future sample; the Beta-Binomial predictive distribution depends on the sample size n and the number of observed successes $\tilde{x}$.

2. For the semiparametric prior, the posterior predictive distribution Is obtained by numerically integrating over the parametric space:

$$p\left(\tilde{x},n\right)=\int_{\Theta }\theta^{\tilde{x}}{\left(1-\theta \right)}^{\left(n-\tilde{x}\right)}f\left(\theta ,m,S,\phi ,y\right)d\theta$$

The integral has been computed via adaptive quadrature over a finite interval, as suggested in the literature [24].

The optimal sample size is the minimum integer among the sample sizes with coverage equal to at least 1−α (or a length at most equal to l for ALC). 

#### 2.2.4. Worst Outcome Optimization

The worst outcome criterion optimizes for both length and coverage; for each sample size, among a subset of x* likelihoods in data space, a Bayesian HPD interval has been estimated. The data in the data subspace have been computed in the Beta-Binomial model as:

$$x^{*}=\{\begin{array}{cc} \frac{n+c+d+1}{2}-c\;\mathrm{or}\frac{n+c+d-1}{2}-c, & \mathrm{if}\;n+c+d\;\mathrm{is}\;\mathrm{odd}\;\mathrm{and}\;n⩾\left|d-c\right|\\ \frac{n+c+d}{2}-c, & \mathrm{if}\;n+c+d\;\mathrm{is}\;\mathrm{even}\;\mathrm{and}\;n⩾\left|d-c\right|\\ n & \mathrm{if}\;0\leq n\leq \left|d-c\right| \end{array}$$

In the previous relation, the values of expected success and failures are, respectively, *c* and *d.*

The x* subspace for a semiparametric setting has been identified by drawing 1000 random samples by B-splines prior before simulating 1000 binomial experiments. The median of the resampled success gives the prior expected successes *c*.

The optimal sample size has been found as the minimum integer ensuring a length of l with coverage equal to at least 1−α. 

The coverages and lengths considered for the sample size computation across all criteria are 1−α=0.95 and l=0.2. The search for the optimal sample size has been performed by considering a grid search around 50% of the frequentist estimate. If the frequentist benchmark sample size is $\hat{n},$ the optimal Bayesian sample size has been identified by searching for the optimization criteria around the integers $\hat{n}-0.5\hat{n}$ and $\hat{n}+0.5\hat{n}$.

### 2.3. Frequentist Sample Size Estimation

A frequentist estimate of sample size for a binomial proportion has also been reported, for comparison purposes, considering an interval length of 0.2 and a confidence level of 0.95. The sample size has been defined by considering a one-arm precision approach tailored to optimize the 95% confidence interval length instead of the statistical power to mimic the phase II trial scenario aimed at estimating a preliminary effect only on a treatment arm. The expected event rate derived from the mean of the expert opinion (0.26) has been considered as a point estimate to derive the sample size. 

### 2.4. Prior Elicitation Procedure

The design of phase II clinical trial aimed to evaluate the effect of oral dexamethasone in reducing kidney scars in infants with a first febrile urinary tract infection (UTI) [25] served as a motivating example.

No objective data were available at the planning stage to define the prior. In this situation, an elicitation experiment has been conducted to obtain a prior distribution of the expert opinion.

An elicitation questionnaire has been submitted to obtain a prior distribution of the probability of observing an adverse event in children with a specific disease. In addition, all the experts involved in the research currently use the treatment in their clinical practice. 

As required in the commonly used SHELF elicitation procedure [26], a joint expert evaluation session was not easy to perform in this research setting. Furthermore, the involvement of several experts simultaneously in the same elicitation session was not easy to implement for practical reasons (related to the different organization of work among experts, the location of work, different shifts, etc.). Therefore, a single expert guessing approach was used; the information was combined among the experts by taking the average of guesses and calculating the variance. This procedure has already been considered in the literature in other contexts [20,27].

Eight opinions $y_{i}$ about the probability of observing an event in pediatric patients was obtained by asking the experts the following question:

*“Based on your experience, what is the probability that a patient aged 0 to 2, with a value of procalcitonin >1 µg/L, treated with the recommended antibiotic regimen, has evidenced the presence of a renal scar event 6 months after the acute episode?”*

The opinions provided by the experts about the probability of a renal scar event were **y** = {0.30, 0.25, 0.15, 0.40, 0.30, 0.20, 0.20, and 0.30}. The mean of the provided opinions is 0.26, and the variance is 0.00625. 

Informative, low-informative, and uninformative prior scenarios are considered for computation using a parametric Beta prior and a semiparametric solution.

### 2.5. Beta Prior Definition

Considering the Beta parametric setting, the strength of prior influence on the final estimation has been defined using a power prior approach [28].

Different levels of penalization (discounting) may be provided on the expert opinion to perform sensitivity analysis on the prior choices. 

The expert opinion may be included in the final computation using a Beta(α,β) prior where:

(1) $$\alpha =\alpha_{0}d_{0}+1\;\beta =\beta_{0}d_{0}+1$$

The $\alpha_{0}$ $\mathrm{and}\;\beta_{0}$ values are the parameters obtained from the mean μ and variance $\sigma^{2}$ of the expert opinions using an inverse formula where:

(2) $$\alpha_{0}=\left[\left(\frac{1-\mu }{\sigma^{2}}-\frac{1}{\mu }\right)\mu^{2}\right]-1\;\beta_{0}=\left[\alpha \left(\frac{1}{\mu }-1\right)\right]-1$$

The value $d_{0}$ defines the amount of expert information to be included in the final result. The discounting factor, otherwise, is defined as $\left(1-d_{0}\right)\times 100$ and represents the levels of penalization (discounting) on the expert opinion.

• If $d_{0}$ = 0, the data provided by the literature are not considered to indicate a 100% discount on prior information. According to this scenario, the prior is an uninformative Beta(1,1) distribution.
• If $d_{0}$ = 1, all the experts’ information is considered in the inference, indicating a 0% discounting of the expert opinion.

### 2.6. B-Spline Prior Definition

The 0.25, 0.5, and 0.75 quartiles have been considered among the expert opinions for the expert elicitation procedure; this is one of the more common approaches to eliciting fractiles [29]. The quantiles have been computed on the vector of the expert opinions **y,** obtaining the following values **α** = {0.2, 0.275, 0.3} of quantiles.

For the semiparametric sample size computation, the strength of influence of the expert opinion on the final result has been defined as varying the ϕ parameter.

**Figure 1:**
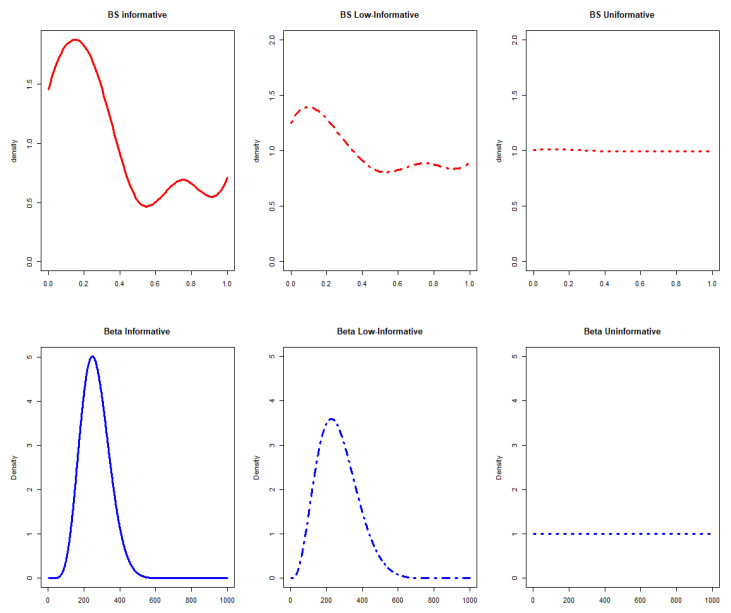
Prior distributions in a parametric (blue graphs) and semiparametric (red graphs) setting considering informative, low-informative, and uninformative scenarios. BS stands for B-splines.

In this general setting, 6 different scenarios were hypothesized for the prior computation (Figure 1).

• Informative priors
  The expert opinions were used to obtain informative prior probability distribution in a parametric or semiparametric setting, considering:
  1. A prior distribution $\;Beta\left(\alpha_{i},\beta_{i}\right)$ with shape and scale obtained from the mean μ and variance $\sigma^{2}$ of the expert opinions, considering $d_{0}$ = 1 (0% discounting) corresponding to Beta(8,22).
  2. A B-splines semiparametric prior defined considering the inner knots located on the expert quartiles with m = 4 degrees of approximation for the B-spline [18]. It has been stated that, by increasing the degrees of approximation, similar results could be obtained with a smoother fit. The prior informativeness parameter, ϕ=0.138, is instead derived from Δ=0.146 as indicated in the literature [18].
• Low-informative priors
  Low-informative priors have been defined in the computation considering:
  1. $Beta\left(\alpha_{i},\beta_{i}\right)$ with a $d_{0}$ = 0.5 (50% discounting) corresponding to Beta(4.5,11.5).
  2. A B-splines semiparametric prior with *m* = 4 degrees and ϕ  = 1.
• Uninformative priors
  Uninformative priors have been compared with other scenarios, respectively in parametric and semiparametric settings deriving the following priors:
  1. A prior distribution *Beta* (1,1) with $d_{0}$ = 0 (100% discounting).
  2. A B-splines semiparametric prior defined considering the inner knots located on the quartiles defined by an expert with *m* = 4 degrees and ϕ = 45.

The prior effective sample size (ESS) has been computed [30,31], and further details are included in the Supplementary Material.

## 3. Results

The frequentist estimated sample size is 75, which is higher than in informative prior scenarios in several cases (Table 1).

The informative and low-informative Beta prior ESS is 30 and 15. The ESS for the B-Spline is 9 and 3, respectively, for the informative and low-informative settings.

Beta informative optimal sample sizes are similar across different estimation methods (GACC, GALC, GWOC) and smaller than samples obtained with other prior distributions, as shown in (Table 1). Generally, the sample sizes are more conservative for GWOC and smaller for the GALC criterion (Table 1). Low-informative sample sizes are a compromise between informative and uninformative scenarios, considering different estimation methods and semiparametric and parametric priors (Table 1).

The estimates provided for an uninformative prior estimation are generally higher than the informative prior results (Table 1). Moreover, the sample size estimates provided for semiparametric B-splines are usually comprised in the sample size derived from the parametric informative and uninformative scenario. A greater variability across results is evidenced in the Beta parametric scenarios compared to B-spline priors. Moreover, a 50% discounting factor ensures similar results compared to a low-informative B-splines prior scenario (ϕ = 1) for ALC and WOC scenarios (Table 1).

**Figure 2:**
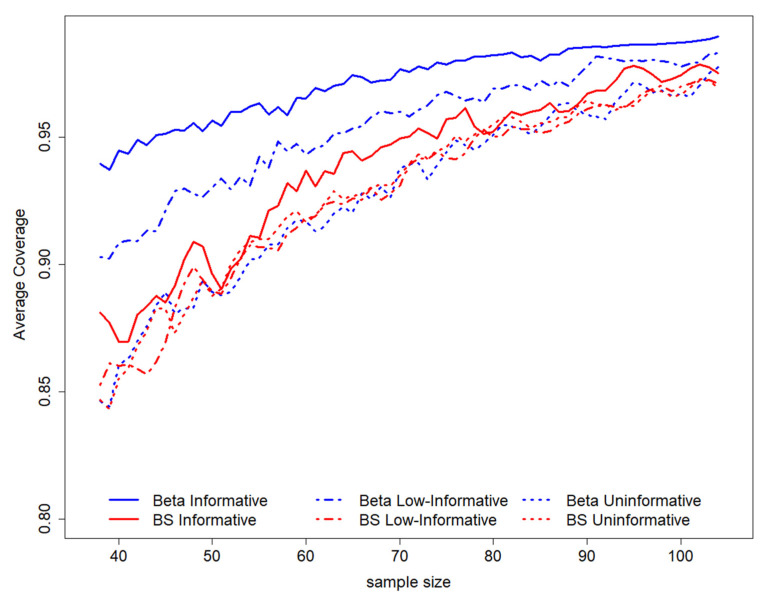
GACC estimation average coverage according to sample sizes for length = 0.2. On the top of the graph, the prior distributions used to perform the study design have been represented. BS stands for B-splines.

By observing the pattern of the average coverage among all possible intervals in the sampling space, according to to sample size (for a fixed length equal to 0.2), it is possible to evaluate a general increase in sample size as the average coverage level increases (Figure 2). The coverage is higher for all sample sizes for a Beta informative prior distribution and is not much different for other scenarios; in this setting, semiparametric results are more similar to the Beta informative scenario (Figure 2).

**Figure 3:**
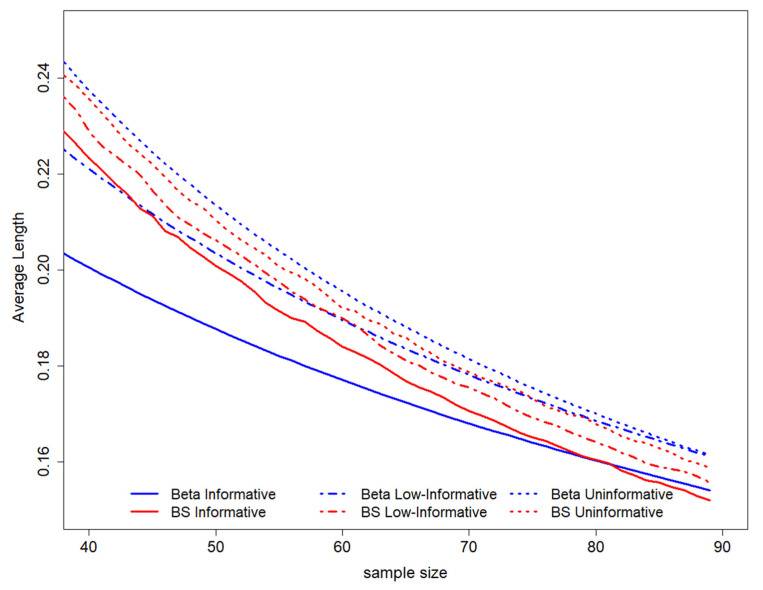
GALC estimation average length according to coverage is equal to 0.95. On the top of the graph, the prior distributions used to perform the study design have been represented. BS stands for B-splines.

When considering the average length among all possible intervals ensuring coverage of 0.95, it is possible to show evidence that this value decreases with decreasing sample size. The higher average length is observed for the uninformative Beta scenario, while the lower average lengths are evidenced for the Beta informative prior. The B-splines elicitation leads to an average length of HPD that lies between the values derived in both Beta scenarios (Figure 3). 

In the phase II study design, an ALC method using informative B-spline priors was selected among scenarios ensuring a sample size of 50 patients. The informative prior was considered to account for the sample size computation information given by the experts about treatment effects, considering that the experts were basically in agreement and have experience with treatment administration. The semiparametric prior was adopted because it considers both the central tendencies of the prior distribution without completely discarding the tail of the expert opinion. The more conventional ALC was selected among sample size methods because 95% coverage is ensured in the data space scenario, regardless of length [32].

However, the computational burden associated with the proposed procedure is not overly high considering that, for B-splines solutions, the optimization problem may be solved with an easy and very fast convergence [18]. 

For GWOC scenarios, the computations were performed considering only the data subspace $x^{*}$ [10]. 

Computations were performed using the R 3.5.2 [33] System with the HDInterval [34], SEL [18], SampleSizeBinomial [35], and LernBayes [36] packages.

## 4. Discussion

Small sample size trials open the way to alternative statistical design and analysis methods. A Bayesian method may be useful when poor accrual problems are evident in clinical research, including prior information about treatment effects, reducing the final estimate uncertainty [37]. In some cases, there may be little objective evidence available, and the expert opinion may be used to elicit an informative prior [29].

The elicitation process may be conducted in parametric, semiparametric, and nonparametric settings, leading to a more flexible approach to translating expert opinions into probability distributions [38]. 

At this point, it would be necessary to use a study design that could consider different approaches to the prior definition. For example, De Santis [39] highlights the importance of a flexible approach to prior distribution in a Bayesian study design considering the possibility of a sample size estimation method that considers different discounting factors defined on the priors, yet without comparing among parametric and nonparametric solutions. 

Especially in study contexts characterized by several sources of uncertainty about the estimated effect and expert opinion, adopting more flexible solutions could be a cue, not only in the design but also in the analysis phase [40]. In such a research field, parametric priors could constrain the expert opinion in a predefined functional form, involving non-conservative small sample sizes for informative prior scenarios. Specifically, concerning the elicitation procedure, the tuning of the parameters defining the distributional form of the prior should be carried out with the guidance of a facilitator in an interactive way, together with the experts involved in the study; the elicited prior distribution should represent a summary of the expert opinions, even if they are conflicting and heterogeneous [26].

The proposed GACC, GALC, and GWOC estimations procedures provide a flexible method to define study designs, taking into account experts’ opinions, and possibly also using nonparametric approaches. 

Compared to a conventional ACC, ALC, and WOC, the proposed methods lead to inclusion in the sample size estimation of alternative prior distributions, compared to a Beta-Binomial framework. 

In phase II clinical trial design, it is possible to observe that the experts may be more or less in agreement about the treatment effect size or may have different experiences or knowledge about the therapy under evaluation. In this context, a more flexible design leads to tailoring the prior distribution and its capability to influence the final results according to the real experts’ knowledge or agreement about the efficacy of the treatment. 

The motivating example presented in this research involves experts who agree about the treatment effect and are skilled in treatment administration in clinical practice. In this framework, a more flexible approach is needed for the prior definition, which must be informative around the expert median and account for the tails of the expert opinion. This leads to more uncertainty in the study design, compared to a Beta informative prior opinion, and greater informativeness compared to a Beta (1,1) prior. 

According to the results of computations, the estimations provided using a generalized approach show more extreme scenarios for sample sizes computed in the parametric framework. The smaller sample sizes are observed for the informative Beta scenario; the semiparametric sample size lies between sample sizes estimated in Beta informative and uninformative settings. Less variability among the results is observed in varying the tuning parameter ϕ across B-splines prior choices, compared to the Beta scenario. Moreover, a ϕ=1 value ensures similar results concerning a parametric solution with a 50% discount for ALC and WOC procedures. 

The B-splines approach guarantees less extreme solutions than a Beta prior, allowing the possibility of achieving sample sizes comparable to results obtained with a Beta prior with a 50% discount (especially for ACC and WOC). 

All of this implies that the generalized method allows for planning a study design with the possibility of considering different kinds of a priori distributions, more or less informative or adapted to experts’ opinions. The choice among priors may depend on confidence in the available information.

Such confidence may depend on the experts’ belief in the treatment effect or the agreement among the experts. Moreover, the methods also give the possibility of using informative parametric methods when objective information is available to derive the prior distributions.

The general framework of results gives solutions for sample size estimation similar to the parametric methods introduced by Joseph [10]. However, the solutions provided by GWOC are more conservative, leading to the simultaneous optimization of the HPD length and coverage.

The sample sizes provided by GACC and GALC are different, resulting in smaller sample sizes for GALC; the same pattern is observed when comparing the ACC and ALC methods proposed by Joseph in a Beta-Binomial framework [10]. Thus, the choice among criteria appears somewhat arbitrary. 

ALC may be considered more convenient because it fixes the coverage, and HPD intervals are computed regardless of length [13]. However, in every case, the choice among criteria averaging over the predictive distribution of the data or considering the worst possible outcome depends on the degree of risk one is willing to take in the final inference [13].

More computational efforts are needed to derive the optimal sample size using the proposed method, compared to the Joseph approach. The computations have been performed by simulating the HPD among all likelihoods in the overall data space (for the ACC and ALC methods) and searching for the sample size around the frequentist estimate. However, the gain regarding flexibility in the study design is considerable, especially for phase II and other studies conducted on small sample sizes.

### Limitations and Future Research Developments

The method may be easily extended by considering a continuous outcome or comparing binary or continuous endpoints across different groups and a wide range of priors in parametric, semiparametric, and nonparametric frameworks. 

The main limitation is that the choice among alternative priors requires evident computational efforts, especially if the posterior is not obtained in closed form or with an easy and fast convergence. More computational time may be required for study designs involving comparisons among groups.

Moreover, the proposed method is based on a semiparametric approach, which is poorly applied in clinical research practice [19]. Further efforts will be needed in this sense to define instruments aimed at facilitating the efficient practical implementation of the proposed design; web applications, graphical supports, and tutorials will be defined to support both the elicitation phase and the subsequent definition of the study design and sample size.

## 5. Conclusions

The generalized solutions to sample size definition provide the possibility of defining a flexible Bayesian experimental design using different prior definitions. Thus, the approach is useful, especially in a clinical trial conducted on small sample sizes and when no objective evidence is available and may be indispensable to summarize expert opinions. The semiparametric sample size solutions are a compromise between the same estimates provided using Beta informative and uninformative methods, ensuring not too different results and varying the tuning parameter ϕ.

When comparing the methods, GWOC is a more conservative solution. However, GALC gives smaller sample sizes compared to GWOC, and the same pattern is observed in the corresponding methods proposed in a Beta-Binomial setting. 

## Figures and Tables

**Table 1:** Optimal sample sizes were defined following GACC, GALC, and GWOC estimation methods using different prior distributions, length = 0.2, coverage = 0.95.

	GACC	GALC	GWOC
Beta Informative	43	42	45
Beta Low-Informative	59	53	76
Beta Uninformative	75	58	92
B-Spline Informative	70	51	71
B-Spline Low-Informative	76	54	77
B-Spline Uninformative	77	56	86

## Data Availability

Not applicable.

## Supplementary Materials

The following supporting information can be downloaded at: https://www.mdpi.com/article/10.3390/ijerph192114245/s1, Figure S1: MC average Difference in Information across sample sizes. The ESS is the integer m minimizing the difference between the prior Information.
