# Supplementary material for: A Bayesian Sample Size Estimation Procedure Based on a B-Splines Semiparametric Elicitation Method
Source: Int J Environ Res Public Health. 2022 Oct 31;19(21):14245. doi: 10.3390/ijerph192114245 (PMC9658653; doi:10.3390/ijerph192114245)
Supplement: Supplementary file 1 [file ijerph-19-14245-s001.zip › ijerph-1980056-supplementary.pdf]

Supplementary Material

Effective Sample Size (ESS)

The prior informativeness, expressed in terms of experimental units, could be calculated via Effective Sample Size (ESS) approach (Neuenschwander et al. 2019). The ESS for the Beta priors has been calculated by summing up the prior parameters α_i and β_i which represent respectively the number of successes and failures(Morita, Thall, and Müller 2008).

The ESS for the semiparametric priors has been computed by applying the generalized idea proposed by Morita(Morita, Thall, and Müller 2008) which extends the ESS concept from conjugate distributions to a generic random variable.

The approach considers the prior information $p(\theta)$ and the Information provided by an uninformative prior $p_0(\theta)$ having the same mean of the $p(\theta)$ prior. The ESS is the integer m that which minimizes the distance between the expected posterior Information for a dataset of size m computed with the $p_0(\theta)$ prior, and the information of the considered $p(\theta)\, prior$ (Morita, Thall, and Müller 2008; Neuenschwander et al. 2020)

The following steps have been considered for the computation:

(1) The prior Information has been computed as the inverse of the prior variance calculated across 1000 resampled BS prior values.
(2) The uninformative BS prior counterpart has been identified by assuming $\phi = 45$.
(3) A sequence of m sample sizes from 1 to 50 has been considered.
(4) For each sample size $m = (1, \ldots, 50)$, a 1000 runs Monte Carlo (MC) experiment has been conducted. Within each MC run, the data have been drawn from a binomial random variable having a probability of success equal to the resampled prior median (Step 1) and a sample size m.
(5) The posterior distributions, for the resampled data within each MC run, have been computed by assuming an uninformative prior as provided in Step 2. The inverse of variance (Information) for each MC resampled posterior has been computed.
(6) The average posterior information has been computed across the 100 MC runs.
(7) The difference between the Prior Information (Step 1) and the Posterior Information (Step 6) has been computed.
(8) The ESS is the sample size m which minimizes the Information difference computed in step 7.

The informative and Low-Informative Beta prior ESS is 30 and 15. The ESS for the BS semiparametric is instead 9 and 3 respectively for the informative and Low-Informative settings (Figure S1).

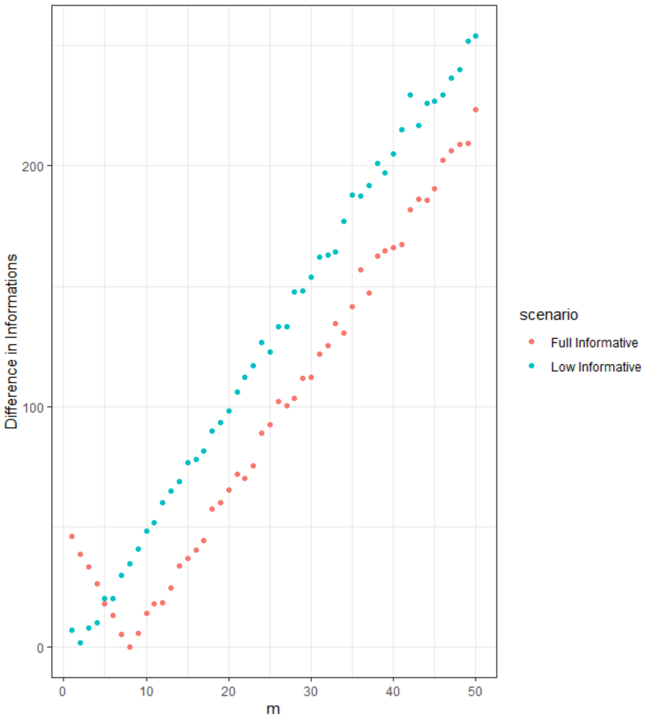

Figure S1. MC average Difference in Informations across sample sizes. The ESS is the integer m minimizing the difference between the prior Information.
